# Assessment of the Effect of Infliximab on Immunomodulation Properties of Mesenchymal Stem Cells *In Vitro*

**DOI:** 10.34172/apb.2021.083

**Published:** 2020-08-05

**Authors:** Yasaman Aliasghari Sakha, Ehsan Ehsani, Elham Roshandel, Arsalan Jalili, Negar Vahdani, Abbas Hajifathali

**Affiliations:** ^1^Developmental Biology, Science and Research Branch, Islamic Azad University, Tehran, Iran.; ^2^Department of Biology, Roudhen Branch, Islamic Azad University, Tehran, Iran.; ^3^Hematopoietic Stem Cell Research Center, Shahid Beheshti University of Medical Sciences, Tehran, Iran.; ^4^Department of Regenerative Medicine, Cell Science Research Center, Royan Institute for Stem Cell Biology and Technology, ACECR, Tehran, Iran.

**Keywords:** Mesenchymal stem cells, Infliximab, Immunomodulation, Tumor necrosis factor

## Abstract

***Purpose:*** Mesenchymal stem cells (MSCs) have immunomodulatory traits making them a promising choice in the treatment of inflammatory diseases such as graft-versus-host disease (GVHD). Tumor necrosis factor-alpha (TNFα) is a major player of inflammatory disease which is blocked by infliximab to reduce the inflammation. The present study aims to assess the infliximab effects on the anti-inflammatory properties of MSCs.

***Methods:*** In this study, bone marrow mesenchymal stem cells (BMMSCs) were co-cultured with peripheral blood mononuclear cells (PBMCs) of GVHD patients in the presence of 10, 20 and 30 µg/mL of infliximab for 48 and 72 hours. The mRNA expression of indoleamine-2,3- dioxygenase (IDO) and inducible nitric oxide synthase (iNOS), as well as the secreted amount of prostaglandin E2 (PGE2) in the culture supernatant, were examined.

***Results:*** The results of this study show that the expression of IDO and iNOS genes, as well as the secretion amount of PGE2 in co-cultured groups raised dramatically, compared to the culture of BMMSCs or PBMCs alone. In co-culture groups containing infliximab, the expression of IDO and iNOS and also the amount of released PGE2 was significantly decreased compared to the control group without infliximab. However, no difference was found in the expression of assayed factors between 48 and 72 hours of treatments.

***Conclusion:*** As an anti-TNFα agent, infliximab can decrease the inflammation in the microenvironment of MSCs, which might mitigate the immunomodulatory effects of MSCs. These effects of anti-inflammatory agents on the immunomodulatory capacity of MSCs should be considered in MSC therapy.

## Introduction

Mesenchymal stem cells (MSCs) are multipotent fibroblast-like cells with differentiation capabilities to various tissues such as adipocytes, chondrocytes, and osteoblasts. Specific characteristics of MSCs, especially immunomodulatory properties, prove that they play a significant role in the process of transplantation.^1^ MSCs reduce the expression of interferon-gamma (IFNγ) and interleukin (IL)-12 by type-1 helper T (Th1) and Th17 cells while stimulating IL-4 secretion from Th2 cells. MSCs cause expansion in CD4^+^/CD25^+^/FoxP3^+^ regulatory T lymphocytes (Tregs), which play an essential role in the immune-tolerance and suppression of pro-inflammatory immune response.^2-4^ MSCs also modulate B lymphocytes proliferation and function (e.g., immunoglobulin production) through both secretory factors and cell-cell contact manner.^5,6^

Infliximab is an approved monoclonal antibody for autoimmune diseases that blocks the interconnection of tumor necrosis factor-alpha (TNFα) with its specific receptors and impede a lot of TNFα-related secondary reactions, including inflammation and leukocytes extravasation.^7-10^ Infliximab specifically blocks TNFα and does not have any impact on TNFβ.^11^

Prostaglandins are inflammatory molecules derived from arachidonic acid during the cyclooxygenase (COX) process, which could be suppressed by COX blockers to reduce the inflammation.^12,13^ Prostaglandin E2 (PGE2) is of the major secretory factors of MSCs regulating the function of immune cells.^14^ Co-culturing immune cells with MSCs reduce the pro-inflammatory cytokine secretion. It has been shown that the anti-inflammation properties of MSCs are dramatically decreased by adding PGE2 blockers into the culture environment, showing the major role of PGE2 in the immunomodulatory effects of MSCs.^14^

Indoleamine-2,3-dioxygenase (IDO) is a rate-limiting enzyme in catalysis of tryptophan, a necessary amino acid for protein synthesis.^15^ Human MSCs express IDO that is a key factor for the suppression of alloreactive T cells responses in mixed lymphocyte reaction.^16^ Under standard in vitro condition, MSCs express negligible amounts of IDO; however, IDO expression in response to the signaling of pro-inflammatory cytokines such as IFNγ and TNFα would be increased remarkably.^16,17^

Nitric oxide (NO) is another MSCs secretory molecule with a paracrine effect which is synthesized by inducible nitric oxide synthase (iNOS). It has been indicated that mouse MSCs suppress T cells through local production of NO.^18^ Moreover, NO produced from MSC induces apoptosis in alloreactive T cells by suppressing STAT-5 phosphorylation. The MSC-derived NO weakens immune responses via induction of T cells apoptosis, thereby enhances the outcome of allotransplantation and prevents graft-versus-host disease (GVHD). Although NO is of important mediators in MSC therapy and can be indicative of MSC function, its short half-life hampers the laboratory measurement of NO.^19^

Nowadays, MSCs are used in treating many inflammatory conditions. One of the most common applications of these cells in cell therapy is for treating patients who suffer from GVHD after allogenic graft of hematopoietic stem cells and do not respond to steroid treatment (as standard treatment).^20,21^ In order to reduce the severity of the disease, the patients receive infliximab as the second phase of therapy. In some cases, patients are under MSCs therapy at the same time as receiving infliximab. Given that these MSCs are influenced by inflammatory cytokine such as TNFα, the aim of this study is the assessment of infliximab effects on MSC immunomodulatory functions through evaluation the IDO, iNOS, and PGE2 expressions in a co-culture of MSCs with allogeneic peripheral blood mononuclear cells (PBMCs) isolated from patients diagnosed with GVHD.

## Methods and Materials

### Isolation and characterization of bone marrow mesenchymal stem cells

The study received approval from the ethics committee of Shahid Beheshti University of Medical Sciences, Tehran, Iran, and all patients and donors signed the informed consent. MSCs were isolated from bone marrow aspiration of healthy donors. These donors were aspirated for ruling out the hematologic malignancies. Ten milliliters of bone marrow sample was aspirated from healthy donors on heparin-coated tubes, then mixed with an equal amount of Dulbecco’s Modified Eagle Medium (DMEM) culture medium. Ten milliliters of the diluted sample was slowly added to 5 mL of ficoll and centrifuged at 450 ×g for 20 minutes. The ring between two isolated phases was collected and washed twice by culture medium, then cultured in cell culture flask containing low glucose DMEM + fetal bovine serum (FBS) 10%. Flask was placed in a humidified incubator at 37°C and 5% CO_2_. The culture medium was replaced every three days for 14 days until the MSCs proliferated and covered the flask. Cells were counted and passaged whenever the confluency reached < 80%. The isolated MSCs were characterized by assessing the expression of surface markers CD44, CD73, CD90 (conjugated with fluorescein isothiocyanate), HLA-DR, CD34, CD45, CD11b (conjugated with phycoerythrin), and CD105 (conjugated with allophycocyanin, all from BD Bioscience, US) using flow cytometry (Attune NXT, Invitrogen, US) as well as adipogenic, chondrogenic and osteogenic differentiation capabilities. All the MSC isolation and characterization were performed based on Delorme et al protocol.^22^

### Co-culture of MSCs with PBMCs

Three patients who diagnosed with GVHD, as an inflammatory disease, were selected for PBMC collection. Ten milliliters of peripheral blood were collected from each patient, and the PBMCs were isolated by centrifugation on the ficoll density gradient, as mentioned above. Bone marrow mesenchymal stem cells (BMMSCs) were seeded with a concentration of 5×10^4^cells/wellon 24-well plates (SPL, South Korea). PBMCs (5×10^5^cells/well) were added to the pre-seeded MSCs after 24 hours. The control wells received nothing more than the PBMCs and MSCs, but in three other groups, infliximab was added to the wells in three different doses (30, 20, and 10 µg/mL). After 48 and 72 hours, the supernatants were collected for ELISA. PBMCs were discarded and MSCs were detached using trypsin-EDTA and washed with phosphate buffer saline (PBS) for quantitative reverse transcriptase polymerase chain reaction (qRT-PCR) analysis.

### RNA isolation and cDNA synthesis

Total RNA was extracted from samples using TRIzol reagent (Life Technologies, Gent, Belgium) according to the manufacturer’s protocol. Briefly, each sample was homogenized in 50μL TRIzol. Twenty-five microliters of chloroform were added to each sample and incubated at room temperature (RT) for 5 minutes. Following the centrifugation at 8000 ×g for 5 minutes, precipitation was performed by adding isopropanol to the collected aqueous phase. The supernatant was removed after centrifugation at 8000 g for 5 minutes at 4°C, and RNA was washed with 80% ethanol. Total RNA was then resuspended in 10 μL DEPC (diethylpyrocarbonate) water and stored at -70°C.

### cDNA synthesis

The concentration of isolated RNA was measured using a spectrophotometer (PicoDrop Real-Life, UK). Complementary DNA (cDNA) was synthesized using PrimeScript QuantiTect Kit (Qiagen, South Korea), and the reaction mixture was prepared following the manufacturer’s instruction. In brief, reactions were carried out in a total volume of the 20 μL reaction mixture containing 2 μL DNA, 13μL total RNA, 4μL enzyme buffer and 1 μL enzyme under the following thermocycling condition: 42ºC for 2 minutes, 42°C for 15 minutes and 95ºC for 3 minutes. The cDNA samples were stored at -20°C until used for the real-time PCR.

### Quantitative reverse transcriptase PCR (qRT-PCR)

Gene expression levels from each sample were determined by qRT-PCR using the Rotor-Gene Q instrument (Qiagen, South Korea). The reactions of qPCR were carried out in a final volume of 10 μL containing 5 μL SYBR Premix Ex Taq II reagent (Takara Bio, US), 0.2 μL of each forward and reverse primers (10μM), 2 μL cDNA template and 2.6 μL of ddH_2_O. The primers used for qPCR are listed in Table 1. The program used for qRT-PCR was an initial denaturation step at 95°C for 1 minutes followed by 50 cycles of denaturation at 95°C for 30 seconds, annealing at 60°C for 30 seconds and extension 72°C for 30 seconds. The melting cycle was started from 72 to 95°C with a 0.2°C/s transition rate.

**Table 1:** Sequence of implemented primers in Real-time PCR

**Gene**	**Primer (5'-3')**	**Size (bp)**
iNOS	Forward: GAGATTGGAGTTCGAGACTTC Reverse : TGGCTAGTGCTTCAGACTTC	125
IDO	Forward: AGAAGTGGGCTTTGCTCTGC Reverse : TGGCAAGACCTTACGGACATCTC	119
ß-actin	Forward: TACCTCATGAAGATCCTCA Reverse : TTCGTGGATGCCACAGGAC	154

iNOS. Inducible nitric oxide synthase; IDO, Indole-amine 2,3-dioxygenase; bp, Base pair.

### ELISA for examining PGE2

The concentration of released PGE2 in the co-culture supernatants was assessed using a human PGE2 ELISA kit (MyBioSource, San Diego, CA, US). Before adding samples/standards, the plate was washed two times. 50 μL of the standards or samples were added to the wells in triplicate. 50 μL of biotin-labeled antibody was added to the wells. The plate was covered and incubated for 45 minutes at 37°C. After three times of wash, 100 μL of Streptavidin-HRP solution was added to each well and incubated for 30 minutes at 37°C. Following five times of wash, 90 μL tetramethylbenzidine-substrate was added to each well and incubated for 20 minutes in the dark. The reaction was stopped by adding 50 μL of stop solution, and the optical density was measured at 450 nm. The concentrations were calculated based on the standard curve.

## Results

### Confirmation of bone marrow mesenchymal stem cells

**Figure 1:**
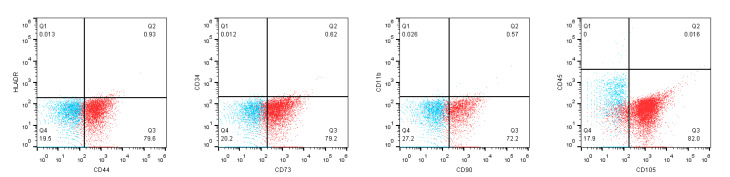
Flow cytometric confirmation of MSCs. Results of flow cytometry analysis on MSCs. Each quadrant represents evaluation of two markers (one positive and one negative marker). The gating strategy is based on isotype control and unstained sample in each quadrant.

The cellular morphology, differentiation ability to chondrocyte, adipocyte and osteocyte, as well as specific surface marker panel were assessed based on Delorme et al protocol to characterize the BMMSCs.^22^ Briefly, the morphology of the cells and their adipogenic, chondrogenic, and osteogenic capacities is identical to a typical MSCs. The results of flow cytometry indicate specific markers expression of MSCs in isolated cells, which confirms the nature of MSCs. These cells are negative in terms of CD34 (0.63%), CD45 (0.01%), CD11b (0.83%) and HLA-DR (0.94%), but express CD44 (80.53%), CD73 (79.82%), CD90 (72.77%) and CD105 (82.01%) markers (Figure 1).

### Effects of infliximab on immunomodulation traits of mesenchymal cells

#### IDO gene expression

**Figure 2:**
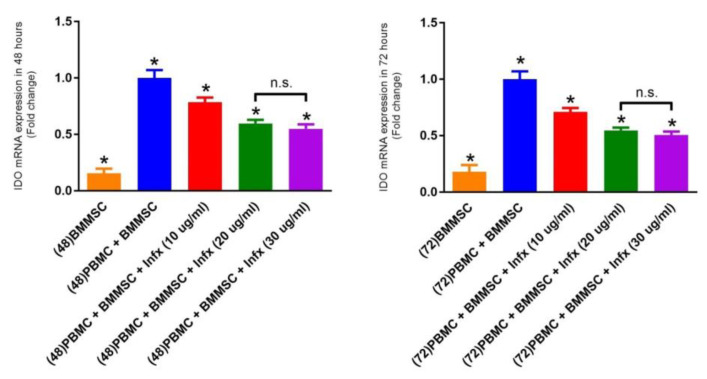
Comparison of IDO gene expression in 48- and 72-hour co-culture. Numbers under the column shows the time of co-culture (48 or 72 hours). Stars on columns represent significant differences between the columns with the others. n.s, not significant; BMMSC, Bone marrow mesenchymal stem cell; PBMC, Peripheral blood mononuclear cell; Infx, Infliximab; IDO, Indole-amine 2,3-dioxygenase.

The expression of the IDO mRNA in BMMSCs alone showed the lowest amount, while the highest IDO expression is seen in those BMMSCs cultured together with PBMCs. Following the co-culture of MSCs with PBMCs in the presence of infliximab, it is shown that the groups containing infliximab have significant lower expression of IDO in BMMSCs, compared to the control group (without infliximab) in both 48 and 72 hours of treatment (Figure 2). Interestingly, as the concentration of the infliximab increased, the expression of IDO mRNA decreased. There is no significant difference in the IDO gene expression between 20 and 30 μg/mL concentrations of treatment. Also, no significant difference is found in IDO gene expression between 48 hours and 72 hours treatment periods.

#### iNOS gene expression

**Figure 3:**
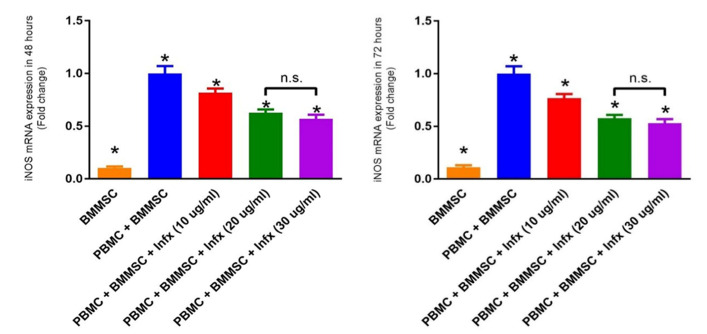
comparison of iNOS gene expression in 48- and 72-hour co-culture. Numbers under the column shows the time of co-culture (48 or 72 hours). Stars on columns represent significant differences between the columns with the others. n.s, not significant; BMMSC, Bone marrow mesenchymal stem cell; PBMC, Peripheral blood mononuclear cell; Infx, Infliximab.

Due to the short half-life of the NO, the gene expression of the iNOS gene was measured to evaluate the capacity of BMMSCs for NO synthesis. As it is demonstrated in Figure 3, the most expression level of iNOS is seen in the co-culture of MSCs with PBMCs without infliximab (control group). In contrast, the least expression was observed in the group in which MSCs are cultured alone without PBMC. After treating with various doses of infliximab, the iNOS gene expression was decreased in a dose-dependent manner. The difference of iNOS gene expression in all three doses is significant compared to control and also each other. The only insignificant change is between the groups treated with 20 and 30 µg/mL of infliximab. Moreover, there was no significant difference in the gene expression of iNOS between the 48 hours and 72 hours culture periods.

#### Examining the amount of PGE2 after encountering with infliximab

**Figure 4:**
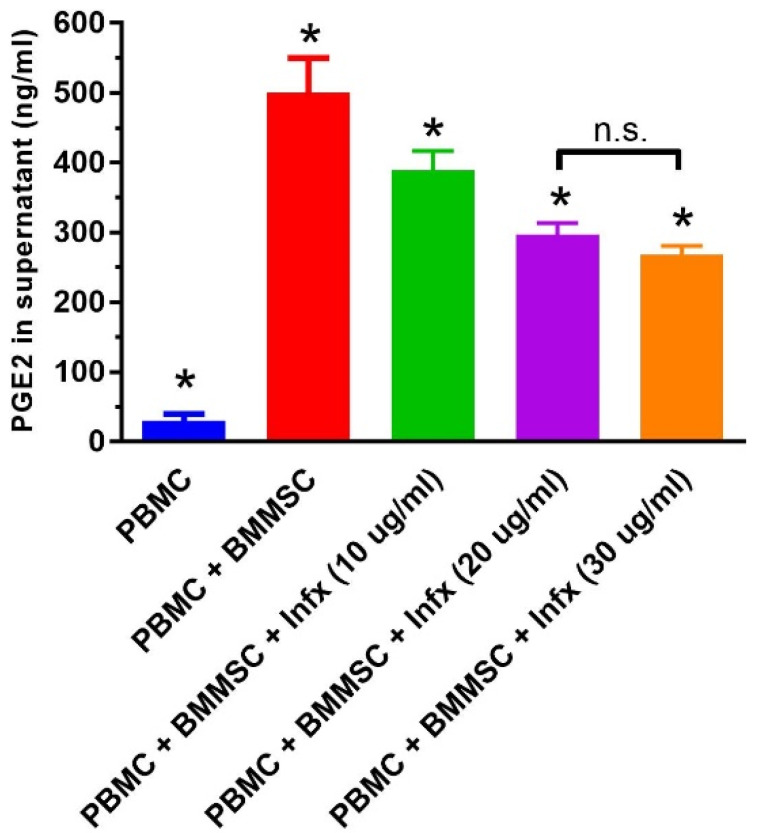
Comparison of PGE2 level in supernatant of 48-hour co-culture. Numbers under the column shows the time of co-culture (48 or 72 hours). Stars on columns represent significant differences between the columns with the others..s, not significant; BMMSC, Bone marrow mesenchymal stem cell; PBMC, Peripheral blood mononuclear cell; Infx, Infliximab; PGE2, Prostaglandin E2.

The level of PGE2 as a main immunomodulatory factor of MSC was examined in the supernatant. The highest amount of PGE2 was observed in the group of PBMC + BMMSC without infliximab. By including and increasing the infliximab in the culture, the PGE2 level decreased significantly. There was no significant difference between PGE2 concentration in the supernatant of groups received 20 and 30 g/mL doses of infliximab treatment (*P* value = 0.69). In this case, regarding the lack of significant differences in the IDO and iNOS genes expression between two different periods of treatment, the level of PGE2 was measured only in the supernatants collected after 48 hours of co-culture. Figure 4 illustrates the examination of the PGE2 level in supernatant culture.

## Discussion

In the present study, the effect of anti-TNFα antibody on the MSCs immunomodulatory functions was shown by co-culturing BMMSCs with PBMCs of GVHD patients as a stimulator. Considering that GVHD is an inflammatory condition that influences different cells of innate and adaptive immunity, PBMCs isolated from these patients can produce inflammatory cytokines affecting MSCs function. Moreover, since the BMMSCs were provided from allogeneic donors, the allogeneic co-culture itself can mimic alloreactive responses and considered a well-known in vitro model of alloreaction and inflammation. The reason for choosing BMMSCs from healthy donors was that the HSCT patients (especially those with GVHD) are in the process of BM homing of stem cells and BM reconstitution. Therefore, they are susceptible to any intervention, especially BM aspiration, which could interfere with homing, engraftment, and immune reconstitution process and lead to inflammation, delayed immune reconstitution, and partial engraftment. It has been proved that the microenvironment is a critical factor in determining the function of MSCs. MSCs often exhibit immunosuppressive effects in highly inflammatory conditions. Stimulation of MSCs through inflammatory signal receptors such as toll-like receptors and cytokine receptors leads to the maximum immunomodulatory function of MSCs.^23^ In other words, in the presence of TNFα, IFNγ, and IL-1β, MSCs could be well-stimulated and presented stronger immunomodulatory features.^23^ It has been suggested that the in vitro treatment of MSCs with pro-inflammatory cytokines can mimic the inflammatory diseases and increase the benefits of MSC therapy.^24^

In line with these studies, we showed that the secretion of PGE2 and gene expression of IDO and iNOS was at the highest level when MSCs were stimulated with GVHD patient-derived PBMCs. Expectedly, these immunomodulatory factors (PGE2, IDO, and iNOS) were at the least level when only MSCs were cultured without external stimulation. Interestingly, in the co-cultures received infliximab, the level of all these immunomodulatory factors were decreased, showing a significant reduction in the immunomodulatory properties of the MSCs. At first glance, due to the anti-inflammatory function of infliximab, it might be expected that the addition of infliximab to the MSCs+PBMCs co-culture should potentiate the immunomodulatory effects of MSCs and synergistically increase the immunosuppression. However, regarding the previous findings, inflammatory signals are necessary for MSCs to stimulate them for maximum immunomodulatory function.^23^

Infliximab is an anti-TNFα monoclonal antibody which blocks the interaction of existent TNFα in the culture medium with TNFα receptors on the MSCs cell surface. As a result, the expression of anti-inflammatory factors by MSCs is decreased. Besides the direct suppressive influence of infliximab on MSCs, infliximab causes the reduction of inflammatory cytokines production from PBMCs, which reduce the PBMCs-mediated stimulation of MSCs, leading to the decrease in immune-regulatory functions of MSCs.^25^ In the non-inflammatory conditions, MSCs express low levels of cyclooxygenase 2, PGE2, TGFβ, and IDO. However, pro-inflammatory conditions can dramatically increase the secretion of anti-inflammatory factors via MSCs. For instance, IFNγ increases the secretion of IDO, HGF, and TGFβ. Moreover, TNFα increases the release of PGE2 by MSCs.^26^ In a study by Aggarwal et al, MSCs were shown to produce small amounts of PGE2 in the culture medium; however, when TNFα was added to the culture medium, PGE2 secretion levels increased dramatically.^3^ Therefore, it could be concluded that a high level of IDO, iNOS, and PGE2 in PBMCs+BMMSCs might be indicative of an inflammatory condition. The reason for evaluating PGE2 is that based on the previously mentioned studies, PGE2 is one of the main immunomodulatory factors of MSCs, which is associated with the production of other anti-inflammatory molecules.^3,14,26^

Another mechanism by which MSCs apply their effects is the release of NO. Nitric oxide is produced by NOS that includes three types; iNOS, endothelial and nervous NOS. NO can suppress T cells proliferation and has the ability to induce apoptosis in T cells. Hence, it could be served as an immunomodulatory factor in alloreactions.^25^ In this study, NO level in supernatant culture was not examined because NO has a short half-life and rapidly metabolizes into NO2 and NO3, so the level of iNOS gene expression was examined instead. MSCs stimulation by IFNγ, and TNFα or IL-1 can induce iNOS. Our data indicate that following MSC co-culture with allogeneic PBMCs in the presence of anti-TNFα antibody, expression of iNOS, as an immunomodulatory sign of MSCs, is reduced and suggest that the presence of inflammatory factors in the microenvironment of MSCs is necessary to induce immunomodulatory function of MSCs which is in line with the result of previous studies.^25-27^

We did not investigate the effects of infliximab on the phenotype, proliferation, and cell cycle of MSCs. However, we did not see any signs of change in terms of morphology and microscopic examination. In previous studies, the effects of infliximab on mouse and human of BMMSCs were examined, and no significant effect of infliximab on the survival, phenotype, morphology, proliferation, cell cycle, and apoptosis of BMMSCs was reported.^27^ It was also suggested that not only the spindle morphology and adhesive ability of MSCs do not change after encountering with infliximab, but also it was revealed that differentiation features of these cells into adipocyte and osteocyte do not change.^27^

Duijvestein et al reported that suppressed proliferation of PBMCs by MSCs still exists in the presence of high concentrations of infliximab, and the neutralization of TNFα alone is not sufficient for the suppression of MSCs effect.^27^ Although we did not investigate the PBMCs proliferation in our study, there seem no significant controversies between what they have found and our findings. Firstly, we reported that by adding an anti-TNFα agent to the co-culture, the immunomodulatory effects of MSCs were decreased but not eliminated. Secondly, we have found that there is no significant difference in assayed factors in groups receiving either 20 or 30 µg/mL of infliximab showing that the high concentration of infliximab cannot further decrease the immunomodulatory properties of MSCs and confirmed the Duijvestein et al finding. On the other hand, it has been shown that besides releasing the immunomodulatory factors, MSCs apply their suppressive effects through cell-cell contact. It has been reported that increased expression of ICAM-1 and VACM-1 in MSCs, strengthens their interaction with T cells.^28^ Thus, the anti-proliferative effect of MSCs on PBMCs that was observed in Duijvestein et al study might be through cell-cell direct contact. We just focused on releasing the immunomodulatory factors of MSCs. A comprehensive examination of the role of anti-TNFα agents on both cell-cell contact capability and immunomodulatory factors of MSCs at the same time can make these ambiguities clear. Moreover, it could be more informative to investigate the other immunosuppressive factors releasing by MSCs both in mRNA and protein forms to find out the exact mechanisms of TNFα on the MSCs function.

## Conclusion

We showed that the presence of infliximab in the co-culture of MSCs with PBMCs of GVHD patients could decrease the expression of anti-inflammatory factors such as IDO, iNOS, and PGE2, which might consequently reduce MSCs modulatory effects. Due to the use of infliximab in inflammatory diseases, the history of infliximab therapy of patients should be taken into account before starting the MSC therapy. In order to prove the effects of anti- or pro-inflammatory drugs on MSC, further investigations are required.

## Ethical Issues

MSCs were isolated from bone marrow aspiration of healthy donors who were suspicious for malignancy and aspirated for ruling out the hematologic malignancies. All patients and donors signed the informed consent, and the study received approval from the ethics committee of Shahid Beheshti University of Medical Sciences, Tehran, Iran (IR.SBMU.RETECH.REC.1398.172).

## Conflict of Interest

The authors declare no conflict of interest.

## Acknowledgments

The authors would like to thank the staff of the Hematopoietic Stem Cell Research Center, Shahid Beheshti University of Medical Sciences, Tehran, Iran for providing the possibility of doing the study and their technical supports.
